# Targeting Microglia–Neuron Crosstalk to Regulate Neuronal Excitability: Novel Translational Approaches for Chronic Pain Intervention

**DOI:** 10.3390/ijms27104622

**Published:** 2026-05-21

**Authors:** Zhenzhen Xu, Yong Lv, Shiqiang Chen, Qingping Wu

**Affiliations:** 1 Department of Anesthesiology, Union Hospital, Tongji Medical College, Huazhong University of Science and Technology, Wuhan 430022, China; 2025xh8026@hust.edu.cn (Y.L.); csq_19880223@126.com (S.C.); 2 Institute of Anesthesia and Critical Care Medicine, Union Hospital, Tongji Medical College, Huazhong University of Science and Technology, Wuhan 430022, China; 3 Key Laboratory of Anesthesiology and Resuscitation, Huazhong University of Science and Technology, Ministry of Education, Wuhan 430022, China

**Keywords:** chronic pain, microglial activation, neuronal excitability, microglia–neuron crosstalk, translational medicine

## Abstract

Chronic pain is a complex and widespread pathological state that severely impairs the quality of life of millions worldwide and imposes a heavy socioeconomic burden. Current therapeutic regimens often fail to provide adequate relief, frequently accompanied by dose-limiting side effects. Emerging evidence suggests that the bidirectional crosstalk between microglia and neurons plays a fundamental role in the development and maintenance of chronic pain. This interaction contributes to central sensitization and enhanced neuronal excitability. This review elucidates the molecular mechanisms underlying microglia–neuron communication. with particular emphasis on its modulation of neuronal excitability. We also discuss innovative translational strategies such as gene therapy, cell therapy, and nanomedicine. Modulating these neuroimmune interfaces represents a promising frontier for developing more precise and efficacious analgesic interventions.

## 1. Introduction

Chronic pain, defined as persistent pain lasting more than 3 months or exceeding the normal tissue healing cycle, is a global public health challenge affecting approximately 20–30% of the adult population worldwide [1,2]. Unlike acute pain, which serves a protective warning function, chronic pain evolves into a pathological state characterized by abnormal neuronal excitability and neuroimmune dysregulation, severely compromising patients’ physical function, mental health, and quality of life [3,4]. It not only causes direct suffering to individuals but also imposes a substantial socioeconomic burden on healthcare systems due to long-term medical expenses, lost productivity, and comorbidities such as anxiety, depression, and cognitive impairment [5]. The pathogenesis of chronic pain is highly complex, involving intricate crosstalk between the nervous and immune systems. In recent decades, research has shifted from the traditional “neuron-centric” perspective to a more comprehensive neuroimmune regulatory framework, revealing that the bidirectional interaction between microglia (the resident immune cells of the central nervous system, CNS) and neurons is the core driver of chronic pain initiation and maintenance [6,7]. Microglial activation and abnormal neuronal excitability are two key pathological features of chronic pain, and their mutual regulation and imbalance disrupt the homeostasis of the neuroimmune network, leading to persistent pain hypersensitivity and abnormal pain perception [8].

Traditional drugs such as non-steroidal anti-inflammatory drugs (NSAIDs) and opioids have little efficacy in neuropathic pain, and the core reason lies in the inaccurate understanding of its pathogenesis [9]. In recent years, neuroimmune crosstalk has become a research focus. As resident immune cells of the central nervous system, microglia have been confirmed to be a key link in the occurrence and maintenance of chronic pain through their bidirectional interaction with neurons [10]. Microglia affect neuronal function by releasing inflammatory factors and regulating synaptic plasticity, while neurons regulate the activation and polarization of microglia through signaling molecules [11,12]. The imbalance of the complex regulatory network formed by the two is the core driving force for the chronicization of chronic pain [13]. In addition, chronic pain is closely related to changes in neuroplasticity, psychological factors (depression and anxiety), social stress, etc., forming a vicious circle [14]. Therefore, in-depth clarification of the interaction mechanism between microglia and neurons, and integration of research results in epidemiology, diagnosis, treatment and other aspects are of great significance for the development of new analgesic strategies. This article systematically synthesizes the latest research progress on the crosstalk between microglial activation and neuronal excitability in chronic pain, covering the neuroimmune basis, molecular mechanisms, pathological processes, clinical diagnosis, therapeutic strategies, research controversies, and future translational prospects. By integrating these findings, we aim to provide a comprehensive theoretical framework for basic research and clinical intervention of chronic pain, and to highlight novel translational approaches targeting microglia-neuron crosstalk to regulate neuronal excitability, thereby promoting the clinical transformation of basic research results.

## 2. Neuroimmune Basis of Chronic Pain: Microglia and Neurons as Core Regulatory Nodes

### 2.1. Biological Characteristics of Microglia and Their Role in Neuroimmune Regulation

Microglia are the primary innate immune cells in the CNS, accounting for 5–10% of all glial cells. Under physiological conditions, microglia exist in a resting state, characterized by a small cell body and long, slender processes that continuously monitor the CNS microenvironment [15]. Their main physiological functions include immune surveillance, clearance of apoptotic cells and debris, maintenance of synaptic homeostasis, and regulation of neurogenesis [16]. Resting microglia express low levels of pro-inflammatory factors and high levels of anti-inflammatory factors, contributing to the maintenance of CNS homeostasis [17]. Upon stimulation by peripheral injury, nerve damage, or inflammatory signals, microglia rapidly transition from a resting state to an activated state, accompanied by significant morphological, phenotypic, and functional changes [18]. Activated microglia undergo hypertrophy of the cell body, shortening and thickening of processes, and increased proliferation and migration to the site of injury [19]. Phenotypically, activated microglia can be divided into pro-inflammatory (M1) and anti-inflammatory (M2) phenotypes, although this classification is not absolute and there is significant phenotypic plasticity [20]. M1-type microglia secrete pro-inflammatory factors such as interleukin-1β (IL-1β), tumor necrosis factor-α (TNF-α), and interleukin-6 (IL-6), which promote neuroinflammation and exacerbate neuronal damage [21]. In contrast, M2-type microglia secrete anti-inflammatory factors such as interleukin-10 (IL-10) and transforming growth factor-β (TGF-β), which inhibit neuroinflammation and promote tissue repair [8].

In the context of chronic pain, microglial activation is an early and persistent pathological event. Peripheral injury or inflammation releases damage-associated molecular patterns (DAMPs) and pathogen-associated molecular patterns (PAMPs), which are recognized by pattern recognition receptors (PRRs) on microglia, such as Toll-like receptors (TLRs) and purinergic receptors (P2X4 and P2X7). Activation of these receptors triggers downstream signaling pathways, such as the p38 mitogen-activated protein kinase (MAPK) and nuclear factor-κB (NF-κB) pathways, leading to the expression and release of pro-inflammatory factors and the maintenance of microglial activation [22,23,24]. This persistent activation of microglia is closely associated with the development of chronic pain, as it directly regulates neuronal excitability and synaptic plasticity [25,26,27].

### 2.2. Biological Characteristics of Neurons and Abnormal Excitability in Chronic Pain

Neurons are the core functional units of the nervous system, responsible for the transmission and integration of pain signals. Pain signals are transmitted from peripheral nociceptors to the spinal dorsal horn, then to the brainstem, thalamus, and cerebral cortex, where they are processed and perceived as pain [28]. The excitability of neurons is tightly regulated by ion channels, neurotransmitters, and neuromodulators, and any disruption of this regulation can lead to abnormal neuronal excitability, which is a key pathological mechanism of chronic pain [29]. In chronic pain states, neurons in the pain pathway undergo significant changes in excitability, including increased spontaneous firing rate, decreased threshold for activation, and enhanced synaptic transmission. These changes are collectively referred to as central sensitization, which is the core mechanism underlying persistent pain hypersensitivity and abnormal pain perception. Central sensitization is mediated by multiple factors, including the upregulation of excitatory ion channels (such as sodium channels Nav1.7 and Nav1.8, and calcium channel CaV3.2), the downregulation of inhibitory ion channels (such as potassium channel KCC2), and the imbalance of neurotransmitters (such as increased glutamate release and decreased γ-aminobutyric acid (GABA) release) [30]. Neuronal excitability is also closely regulated by the surrounding microenvironment, especially the crosstalk with microglia [31]. Conversely, neurons can release neurotransmitters and DAMPs, which in turn activate microglia, forming a positive feedback loop that amplifies neuroinflammation and abnormal neuronal excitability [32].

### 2.3. Neuroimmune Network Imbalance in Chronic Pain

The neuroimmune network in the CNS is a complex regulatory system composed of neurons, microglia, astrocytes, and other immune cells, which maintains the homeostasis of the nervous system through mutual interaction [33]. In chronic pain, this network is disrupted, leading to an imbalance between pro-inflammatory and anti-inflammatory responses, and abnormal regulation of neuronal excitability [2]. Microglia and neurons are the core nodes of the neuroimmune network in chronic pain. Activated microglia release pro-inflammatory factors, which not only enhance neuronal excitability but also activate astrocytes, another important type of glial cell in the CNS [34,35]. Activated astrocytes further release pro-inflammatory factors and chemokines, recruiting more microglia to the site of injury and amplifying the neuroinflammatory response [36]. This synergistic activation of microglia and astrocytes forms a neuroinflammatory cascade, which maintains central sensitization and persistent chronic pain [37]. In addition, the neuroimmune network imbalance is also regulated by the peripheral immune system. Peripheral inflammation can lead to the infiltration of immune cells (such as macrophages and T cells) into the CNS, which interact with microglia and neurons to further exacerbate neuroinflammation and abnormal neuronal excitability [2]. This bidirectional communication between the peripheral and central immune systems plays an important role in the initiation and maintenance of chronic pain.

## 3. Molecular Mechanisms of Bidirectional Interaction Between Microglial Activation and Neuronal Excitability in Chronic Pain

### 3.1. Core Carriers and Molecular Mediators of Interaction

**Figure 1:**
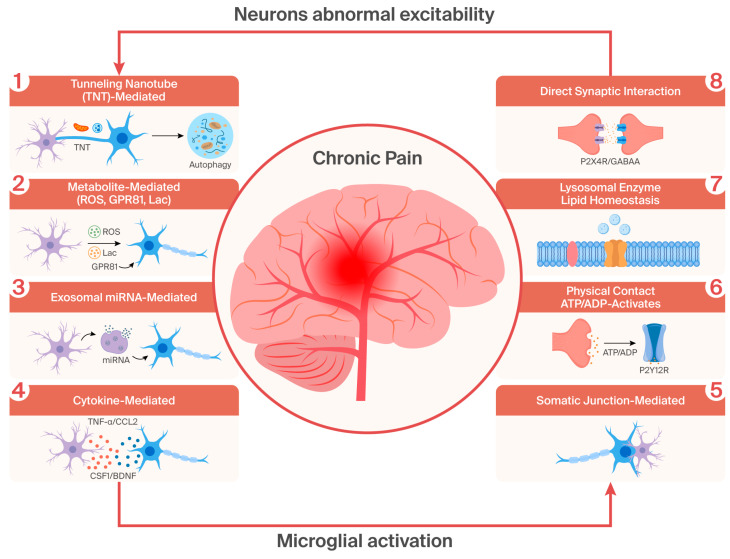
Bidirectional microglia-neuron crosstalk in chronic pain pathogenesis. This schematic summarizes eight key interactive mechanisms underlying microglial activation and neuronal hyperexcitability in chronic pain: Tunneling nanotube (TNT)-mediated transfer: Mitochondria and toxic proteins are exchanged between microglia and neurons to regulate energy homeostasis and protein clearance. Metabolite-mediated signaling: Microglial ROS induces neuronal oxidative damage; neuronal lactate activates microglial GPR81 to promote pro-inflammatory cytokine release. Exosomal miRNA-mediated regulation: Microglial and neuronal exosomal miRNAs modulate neuronal excitability and microglial polarization. Cytokine-mediated signaling: Microglial cytokines (TNF-α, BDNF) sensitize nociceptive neurons, while neuronal chemokines (CCL2, CSF1) drive microglial activation. Somatic junction-mediated interaction: Direct physical contacts facilitate synaptic pruning and mitochondrial status sensing. Physical contact-mediated signaling: Neuronal ATP/ADP activates microglial P2Y12R to guide process migration. Lysosomal enzyme/lipid homeostasis: Microglial β-hexosaminidase maintains neuronal lipid stability via GM2 ganglioside degradation. Direct synaptic interaction: Microglial MMPs remodel perineuronal nets; neuronal ATP/GABA bidirectionally regulates microglial activation. These pathways form a self-amplifying cycle that sustains central sensitization and chronic pain.

The interaction between microglia and neurons relies on specific molecular mediators and structural carriers, forming a multi-dimensional signal transduction network, where each mediator undertakes a bidirectional regulatory function (Figure 1):

#### 3.1.1. Cytokine-Mediated Bidirectional Interaction

**Figure 2:**
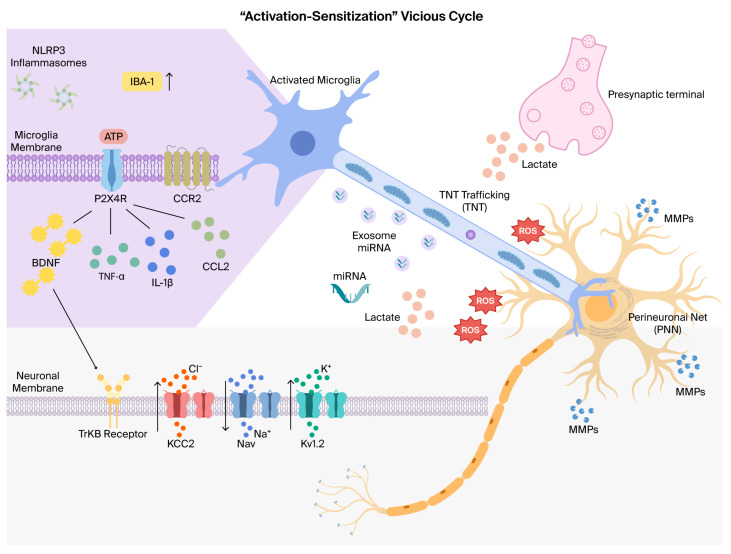
The “activation-sensitization” vicious cycle of microglia-neuron crosstalk in chronic pain. Microglial activation and inflammatory signaling: Pathological stimuli induce microglial activation, characterized by the marked upregulation of IBA-1 and the assembly of NLRP3 inflammasomes. Stimulated by ATP, microglial P2X4 receptors (P2X4R) trigger the release of a cascade of pro-nociceptive mediators, including the neurotrophin BDNF, pro-inflammatory cytokines (IL-1β, TNF-α), and the chemokine CCL2 (acting via the CCR2 receptor). Neuronal hyperexcitability and ion channel remodeling: Microglial-derived BDNF activates neuronal TrkB receptors, leading to the functional downregulation of the potassium-chloride cotransporter KCC2. This disruption of chloride (Cl^−^) homeostasis impairs inhibitory neurotransmission. The neuroinflammatory milieu modulates voltage-gated ion channels, specifically enhancing Nav channel activity and altering Kv1.2 channel conductance, which collectively lower the neuronal firing threshold and promote neuron sensitization. Alternative intercellular communication and matrix remodeling: Beyond paracrine signaling, cells communicate via tunneling nanotubes (TNTs) for the direct trafficking of organelles (e.g., mitochondria) and exosomes for the horizontal transfer of miRNA. The accumulation of metabolic byproducts, such as lactate and reactive oxygen species (ROS), further fuels the inflammatory environment. Additionally, microglial-secreted matrix metalloproteinases (MMPs) catalyze the degradation of the perineuronal net (PNN), an extracellular matrix structure that stabilizes synapses, thereby facilitating maladaptive synaptic plasticity. Long arrows indicate ion transport across cell membranes or receptor-ligand binding, whereas short arrows represent elevated IBA-1 expression levels. Figure 2 was drawn by Figdraw (www.figdraw.com, accessed on 13 April 2026).

Microglia-to-neuron crosstalk is mediated by numerous molecular pathways: Activated microglia release pro-inflammatory factors such as TNF-α and IL-1β, which bind to corresponding receptors on the neuronal surface, downregulate the expression of potassium channels (e.g., Kv1.2), and enhance the activity of sodium channels (Nav1.7/1.8) and calcium channels (TRPV1), thereby significantly increasing neuronal excitability [38,39,40]. The released BDNF binds to the TrkB receptor on neurons, altering the neuronal anion gradient, inhibiting GABAergic inhibitory synaptic transmission, and enhancing pain signal conduction. In the SNI mouse model, knockout of the BDNF gene in microglia can significantly increase the paw withdrawal threshold and alleviate mechanical allodynia [41,42,43]. Neuron-microglia crosstalk may be mediated by numerous intrinsic signaling pathways: CCL2 and CSF1 released by neurons can specifically bind to CCR2 and CSF1R receptors on the microglial surface, inducing microglial activation, proliferation, and polarization toward the M1 phenotype [44]. In contrast, CX3CL1 released by neurons under physiological conditions binds to CX3CR1 on microglia, maintaining the resting phenotype of microglia and inhibiting excessive activation [6]. The level of CCL2 in the cerebrospinal fluid of patients with chronic low back pain is positively correlated with the microglial activation marker (IBA-1) (Figure 2).

#### 3.1.2. Direct Synaptic Interaction

There exist multiple potential pathways underlying microglia to neurons crosstalk: Under physiological conditions, microglia contact synapses through processes and clear excess or abnormal synapses through phagocytic function, helping to precisely shape neuronal circuits [42]. Under pathological conditions, activated microglia release matrix metalloproteinases (MMPs), which degrade the perineuronal net (PNN)—an extracellular matrix structure surrounding nociceptive projection neurons—removing its regulatory restrictions on neurons, enhancing neuronal activity, and inducing pain hypersensitivity [45]. Neuron-microglia crosstalk may be mediated by numerous intrinsic signaling pathways: ATP released from neuronal synapses can bind to P2X4 receptors on the microglial surface, activate the downstream ERK/p38 MAPK pathway, and promote the release of pro-inflammatory factors [46]. In contrast, GABA released from the presynaptic membrane can inhibit excessive activation of microglia through the GABAA receptor on microglia, forming a negative feedback regulation [47]. In the CCI model, blocking neuronal ATP release can significantly reduce the expression of P2X4 receptors in microglia and alleviate hyperalgesia [48].

#### 3.1.3. Exosome/miRNA-Mediated Indirect Interaction

Multiple regulatory pathways have been implicated in neuron-to-microglia intercellular crosstalk: Exosomes released by neurons carry miR-124, which can be phagocytosed by microglia and then target and inhibit the TLR4/NF-κB pathway, reducing the production of pro-inflammatory factors and maintaining the resting state of microglia [49]. Under chronic pain conditions, the secretion of miR-124 by neurons decreases, and the inhibitory effect on microglia is weakened [50]. Several putative signaling axes govern microglial modulation of neuronal function: Exosomes released by microglia contain miR-26a-5p, which can be taken up by neurons and target the MAPK6 gene, inducing abnormal neuronal discharge and spontaneous pain. Clinical studies have shown that the level of microglia-derived miR-26a-5p in the serum of patients with neuropathic pain is significantly increased [51]. Pro-inflammatory factors released by activated microglia are key mediators of the bidirectional interaction between microglial activation and neuronal excitability [52]. IL-1β, TNF-α, and IL-6 are the most extensively studied pro-inflammatory factors in chronic pain, and they exert significant regulatory effects on neuronal excitability through multiple pathways [53]. IL-1β is a key pro-inflammatory factor that is highly expressed in activated microglia in chronic pain models. It can bind to IL-1 receptors on neurons, activating downstream signaling pathways such as the MAPK and phosphatidylinositol 3-kinase (PI3K)/Akt pathways, which upregulate the expression of excitatory ion channels (such as Nav1.7 and CaV3.2) and downregulate the expression of inhibitory ion channels (such as KCC2), thereby increasing neuronal excitability [54,55]. In addition, IL-1β can promote the release of glutamate from neurons, enhancing synaptic transmission and further amplifying central sensitization [56]. Conversely, neurons can release ATP and other DAMPs upon activation, which bind to P2X4 and P2X7 receptors on microglia, triggering the release of IL-1β and promoting microglial activation, forming a positive feedback loop between microglial activation and neuronal excitability [24,57,58]. TNF-α is another important pro-inflammatory factor that is closely associated with chronic pain. It can bind to TNF receptors on neurons, activating the NF-κB pathway, which upregulates the expression of pro-inflammatory genes and excitatory ion channels, increasing neuronal excitability [59]. TNF-α can also induce the apoptosis of inhibitory neurons, reducing the inhibitory effect on pain-signaling neurons and further enhancing central sensitization [60]. In addition, TNF-α can promote the activation of microglia and astrocytes, amplifying the neuroinflammatory response [61]. IL-6 is involved in the regulation of microglia–neuron crosstalk by promoting microglial activation and enhancing neuronal excitability [62]. It can bind to IL-6 receptors on microglia, activating the JAK/STAT3 pathway, which upregulates the expression of pro-inflammatory factors and promotes microglial activation [53]. On the other hand, IL-6 can act on neurons to upregulate the expression of Nav1.8 channels, increasing their excitability and promoting the transmission of pain signals [60,63].

### 3.2. Purinergic Signal-Mediated Regulation

Purinergic signaling, mediated by ATP and its metabolites, is an important pathway for the bidirectional interaction between microglial activation and neuronal excitability in chronic pain. ATP is released by damaged neurons and glial cells as a DAMP, and can be recognized by purinergic receptors on microglia and neurons, triggering a series of signaling events [64]. P2X4 and P2X7 receptors are the main purinergic receptors expressed on microglia. Activation of P2X4 receptors by ATP promotes the release of brain-derived neurotrophic factor (BDNF) from microglia [65]. BDNF binds to TrkB receptors on neurons, downregulating the expression of KCC2, which disrupts the chloride ion homeostasis of neurons, weakens GABAergic inhibitory synaptic transmission, and increases neuronal excitability. This mechanism is a key molecular basis for central sensitization in neuropathic pain [30]. Neurons also express purinergic receptors, such as P2X3 and P2Y receptors, which are involved in the regulation of neuronal excitability [66]. ATP released by microglia can bind to P2X3 receptors on nociceptive neurons, increasing their excitability and promoting the transmission of pain signals [67]. In addition, ATP metabolites such as adenosine can bind to adenosine receptors on microglia and neurons, exerting anti-inflammatory and analgesic effects by inhibiting microglial activation and reducing neuronal excitability, which provides a potential target for chronic pain treatment.

### 3.3. Neurotrophic Factor-Mediated Regulation

Neurotrophic factors, such as BDNF, nerve growth factor (NGF), and glial cell line-derived neurotrophic factor (GDNF), play an important role in the bidirectional interaction between microglial activation and neuronal excitability [68]. BDNF is the most extensively studied neurotrophic factor in chronic pain, and it is mainly released by activated microglia and neurons [69]. As mentioned earlier, BDNF released by activated microglia binds to TrkB receptors on neurons, downregulating KCC2 expression and increasing neuronal excitability [70]. In addition, BDNF can promote the proliferation and activation of microglia by binding to TrkB receptors on microglia, forming a positive feedback loop [71]. NGF is mainly released by peripheral tissues and immune cells, and it can bind to TrkA receptors on nociceptive neurons, promoting their survival and increasing their excitability [72]. NGF can also induce the activation of microglia, promoting the release of pro-inflammatory factors and further enhancing neuronal excitability [73]. GDNF, on the other hand, exerts analgesic effects by inhibiting microglial activation and reducing neuronal excitability [74], which provides a potential therapeutic target for chronic pain.

## 4. Pathological Mechanisms of Chronic Pain: Focus on Microglia–Neuron Crosstalk

### 4.1. Inducing Factors of Chronic Pain

Chronic pain can be induced by a variety of factors, including peripheral nerve injury, tissue inflammation, cancer, diabetes, and psychological stress [59]. These factors trigger the activation of microglia and abnormal neuronal excitability through different pathways, leading to the development of chronic pain [60]. Peripheral nerve injury (such as nerve transection, ligation, or crush) is a common cause of neuropathic pain. Nerve injury leads to the release of DAMPs (such as ATP and HMGB1) and neurotransmitters (such as glutamate), which activate microglia in the spinal dorsal horn [64]. Tissue inflammation (such as rheumatoid arthritis, inflammatory bowel disease) is another common cause of chronic pain. Inflammatory mediators (such as prostaglandins and cytokines) released by inflamed tissues activate peripheral nociceptors and trigger the infiltration of immune cells into the CNS, leading to the activation of microglia and abnormal neuronal excitability [75,76]. This neuroinflammatory response maintains central sensitization and persistent inflammatory pain. Cancer-induced pain is a complex type of chronic pain, involving both inflammatory and neuropathic mechanisms. Tumor cells release pro-inflammatory factors and growth factors, which activate microglia and neurons, leading to abnormal neuronal excitability and persistent pain [74,77]. In addition, cancer treatment (such as chemotherapy and radiation therapy) can also cause nerve damage, further exacerbating chronic pain [78]. Diabetes is a common metabolic disease that can cause diabetic peripheral neuropathy, a major cause of chronic pain [79]. Hyperglycemia leads to oxidative stress and neuroinflammation, activating microglia and inducing abnormal neuronal excitability [80]; this pathological process leads to the loss of peripheral nerves and persistent pain. Psychological stress (such as anxiety, depression or chronic stress) can also induce or exacerbate chronic pain. Stress activates the hypothalamic–pituitary–adrenal (HPA) axis, leading to the release of glucocorticoids, which promote microglial activation and abnormal neuronal excitability [81]. In addition, stress can disrupt the balance of the neuroimmune network, further amplifying the pathological process of chronic pain.

### 4.2. Central Sensitization: Core Pathological Process Mediated by Microglia–Neuron Crosstalk

Central sensitization is the core pathological process of chronic pain, characterized by increased excitability of pain-signaling neurons in the CNS, leading to pain hypersensitivity, allodynia, and spontaneous pain [82]. Microglia–neuron crosstalk plays a key role in the initiation and maintenance of central sensitization [83]. In the early stage of pain injury, peripheral injury signals are transmitted to the spinal dorsal horn, activating microglia [84]. This leads to the initiation of central sensitization. In the chronic stage, persistent microglial activation and neuroinflammation maintain the abnormal excitability of neurons, leading to the persistence of central sensitization [85,86,87]. Activated microglia release chemokines (such as CXCL1 and CCL2), which recruit and activate astrocytes [88,89]. This synergistic interaction between microglia and neurons forms a neuroinflammatory cascade that maintains persistent central sensitization and chronic pain.

## 5. Clinical Diagnosis of Chronic Pain

The clinical diagnosis of chronic pain is mainly based on the patient’s subjective symptoms, clinical manifestations, and auxiliary examinations, and there is currently no specific biological marker for accurate diagnosis [80]. The diagnosis process needs to clarify the type, location, intensity, duration, and triggering factors of pain, as well as the impact of pain on the patient’s quality of life and comorbidities [81].

### 5.1. Clinical Assessment Tools

Subjective pain assessment tools are the main methods used for the clinical diagnosis of chronic pain. The Visual Analog Scale (VAS), Numerical Rating Scale (NRS), and Verbal Rating Scale (VRS) are commonly used to assess pain intensity [90]. The VAS uses a 10 cm line, with 0 representing no pain and 10 representing the most severe pain, and the patient marks the corresponding position according to their pain experience. The NRS uses numbers 0–10 to assess pain intensity, with 0 representing no pain and 10 representing the most severe pain [91]. The VRS uses verbal descriptions (such as no pain, mild pain, moderate pain and severe pain) to assess pain intensity. In addition, the Pain Disability Index (PDI) and Short Form-36 (SF-36) are used to assess the impact of pain on the patient’s physical function, mental health, and quality of life [92,93]. SF-36 assesses the patient’s physical and mental health status from eight dimensions, namely physical function, role physical, bodily pain, general health, vitality, social function, role emotional, and mental health.

### 5.2. Auxiliary Examinations

Auxiliary examinations are used to exclude other diseases and confirm the pathological basis of chronic pain. Neurophysiological examinations, such as electromyography (EMG), nerve conduction velocity (NCV), and evoked potentials, are used to assess nerve damage and abnormal neuronal excitability in chronic pain [89]. For example, NCV can detect the speed of nerve conduction, and abnormal NCV indicates nerve damage, which is helpful for the diagnosis of neuropathic pain [90]. Imaging examinations, such as magnetic resonance imaging (MRI), computed tomography (CT), and positron emission tomography (PET), are used to assess the structure and function of the CNS and peripheral nerves [92]. MRI can clearly show the structure of the spinal cord, brain, and peripheral nerves, and detect abnormalities such as nerve compression, inflammation, and tumor [94]. PET can detect the metabolic activity of the brain and spinal cord, and abnormal metabolic activity in pain-related brain regions (such as the thalamus or cerebral cortex) indicates abnormal neuronal activity, which is helpful for the diagnosis of chronic pain [93]. Biological marker detection is a promising direction for the diagnosis of chronic pain. Studies have shown that the levels of pro-inflammatory factors (IL-1β, TNF-α and IL-6) in the cerebrospinal fluid and peripheral blood of chronic pain patients are significantly increased. In addition, the levels of BDNF and purine metabolites (such as ATP) are also abnormal. These biological markers may provide a basis for the objective diagnosis of chronic pain, but further research is needed to confirm their clinical value [95].

## 6. Therapeutic Strategies for Chronic Pain: Targeting Microglia–Neuron Crosstalk

The current clinical treatment of chronic pain mainly focuses on relieving pain symptoms, but there is no cure. The main therapeutic strategies include drug therapy, physical therapy, psychological therapy, and surgical therapy [96]. With the in-depth understanding of the role of microglia–neuron crosstalk in chronic pain, targeted therapeutic strategies based on this mechanism have become a research hotspot in translational medicine [6] (Table 1).

### 6.1. Drug Therapy Targeting Microglial Activation

Drug therapy targeting microglial activation mainly aims at inhibiting microglial activation and reducing the release of pro-inflammatory factors, thereby alleviating neuroinflammation and abnormal neuronal excitability. Non-steroidal anti-inflammatory drugs (NSAIDs) are commonly used to inhibit neuroinflammation by blocking the synthesis of prostaglandins, but their long-term use has significant side effects (such as gastrointestinal or renal damage) [97,98]. Minocycline, a tetracycline antibiotic, has been shown to inhibit microglial activation by blocking the p38 MAPK and NF-κB pathways, reducing the release of pro-inflammatory factors and alleviating chronic pain [99]. Clinical trials have shown that minocycline can effectively relieve neuropathic and inflammatory pain, with good safety [100]. P2X4 and P2X7 receptor antagonists are another class of drugs targeting microglial activation. TNP-ATP, a P2X4 receptor antagonist, can inhibit the activation of P2X4 receptors on microglia, reduce the release of BDNF, and alleviate central sensitization and chronic pain [101,102]. These drugs are currently in preclinical or clinical trials, and their clinical efficacy needs to be further confirmed.

### 6.2. Drug Therapy Targeting Neuronal Excitability

Drug therapy targeting neuronal excitability mainly aims to regulate the expression and function of ion channels on neurons, thereby reducing abnormal neuronal excitability and relieving pain. Sodium channel blockers (such as carbamazepine, gabapentin, pregabalin) are commonly used in the treatment of chronic pain [103,104]. Gabapentin and pregabalin bind to the α2δ subunit of calcium channels, reducing the release of glutamate and inhibiting neuronal excitability, which can effectively relieve neuropathic pain [105,106]. Potassium channel activators (such as retigabine) can activate potassium channels on neurons, increasing potassium ion outflow, hyperpolarizing neurons, and reducing their excitability [107]. Clinical trials have shown that retigabine can effectively relieve neuropathic pain, but its use is limited by side effects such as dizziness and somnolence [108]. NMDA receptor antagonists (such as ketamine) can block NMDA receptors on neurons, inhibit glutamate-mediated excitatory synaptic transmission, and reduce neuronal excitability [109,110]. Ketamine has been used in the treatment of refractory chronic pain, but its use is limited by side effects such as hallucinations and hypertension [111].

### 6.3. Combined Therapy Targeting Microglia–Neuron Crosstalk

Due to the complexity of chronic pain pathogenesis, single-drug therapy often has limited efficacy. Combined therapy targeting microglia–neuron crosstalk has become a new direction for chronic pain treatment [112]. For example, combining minocycline (inhibiting microglial activation) with gabapentin (inhibiting neuronal excitability) can synergistically alleviate chronic pain by targeting both microglial activation and neuronal excitability [113]. In addition, combining drug therapy with physical therapy, psychological therapy, or acupuncture can further improve the therapeutic effect of chronic pain. Physical therapy (such as massage or electrical stimulation) can improve local blood circulation, relieve muscle spasm, and reduce pain [114]. Psychological therapy (such as cognitive–behavioral therapy or mindfulness therapy) can help patients adjust their psychological state, reduce the impact of pain on their quality of life, and enhance the efficacy of drug therapy [115]. Acupuncture can regulate the neuroimmune network, inhibit microglial activation, and reduce neuronal excitability, thereby relieving chronic pain [116].

### 6.4. Novel Translational Therapeutic Approaches

With the advancement of translational medicine, novel therapeutic approaches targeting microglia-neuron crosstalk have become promising for chronic pain intervention, as abnormal bidirectional communication between microglia and neurons mediates central sensitization in chronic pain [117]. Conventional therapies lack specificity and have limited efficacy, making targeted strategies necessary. Novel therapeutic approaches targeting microglia-neuron crosstalk have been continuously developed, including gene therapy, cell therapy, and nanomedicine [118]. Gene therapy achieves precise molecular intervention by delivering targeted gene vectors (e.g., adeno-associated virus, lentivirus) to regulate the expression of key functional genes in microglia and neurons, thereby restoring the homeostasis of their interaction. For example, overexpression of interleukin-10 (IL-10), a critical anti-inflammatory factor, in microglia can effectively inhibit the activation of the NF-κB pro-inflammatory signaling pathway, reduce the release of pro-inflammatory cytokines, and block neuroinflammation-induced neuronal hyperexcitability, ultimately exerting significant analgesic effects in animal models of chronic neuropathic pain [119]. Additionally, RNA interference (RNAi) technology can silence pro-inflammatory genes (e.g., TLR4 and CX3CR1) in microglia or excitatory genes (e.g., Nav1.7 and GluN2B) in neurons, providing a complementary approach to disrupt abnormal microglia-neuron crosstalk and relieve pain[k1]. Cell therapy, primarily using mesenchymal stem cells (MSCs), exerts analgesic effects by leveraging their immunomodulatory and neuroprotective properties [120]. MSCs secrete a variety of anti-inflammatory factors (e.g., IL-10 and TGF-β) and neurotrophic factors (e.g., BDNF and GDNF), which not only inhibit the M1 polarization of microglia (pro-inflammatory phenotype) and promote their transformation to the M2 phenotype (anti-inflammatory and repair phenotype) but also enhance neuronal survival and synaptic plasticity, thereby reversing neuronal hyperexcitability [121]. Clinical trials have further confirmed the safety and efficacy of MSC transplantation: a phase II trial involving 74 patients with chronic neuropathic pain showed that MSC therapy significantly reduced visual analog scale (VAS) scores and improved quality of life without severe adverse reactions [122]. Moreover, stem cell-derived extracellular vesicles (EVs) have recently attracted attention, as they can carry regulatory molecules to target microglia and neurons, avoiding the potential risks of direct stem cell transplantation and showing great translational potential [120]. Nanomedicine addresses the bottlenecks of traditional drug therapy (e.g., poor bioavailability, low targeting and systemic side effects) by using nanocarriers (e.g., liposomes and polymeric nanoparticles) for targeted drug delivery [123,124]. For instance, CD11b antibody-modified nanocarriers loaded with minocycline can specifically target activated microglia in the spinal dorsal horn, inhibit microglial activation and neuroinflammation, and achieve long-term analgesia without obvious systemic toxicity [125]. For example, nanocarriers loaded with minocycline can specifically target microglia, inhibit microglial activation, and relieve chronic pain [126]. Similarly, gabapentin-loaded nanocarriers modified with neuronal surface markers (e.g., Thy1.1) can specifically bind to neurons, reduce the expression of excitatory ion channels, and improve the therapeutic effect of chronic neuropathic pain [127]. These novel translational therapeutic approaches provide new ideas for the treatment of chronic pain, but further research is needed to promote their clinical application.

**Table 1:** Therapeutic Strategies for Chronic Pain.

Therapeutic Category	Specific Strategy	Representatives	Mechanism	Characteristics/ Limitations	References
Pharmacological Therapy Targeting Microglial Activation	Inhibit microglial activation and neuroinflammation	NSAIDs	Block prostaglandin synthesis	Long-term use: gastrointestinal/renal injury	[97,98]
		Minocycline	Block p38 MAPK/NF-κB pathways	Effective, favorable safety	[99]
		P2X4 antagonist (TNP-ATP)	Inhibit P2X4, reduce BDNF	Preclinical/clinical; efficacy to confirm	[101,102]
		P2X7 antagonist (BBG)	Inhibit NLRP3, reduce IL-1β	Preclinical/clinical; efficacy to confirm	[128]
Pharmacological Therapy Targeting Neuronal Excitability	Regulate ion channels, reduce excitability	Carbamazepine	Bind calcium	First-line for neuropathic pain	[103,105]
		gabapentin	channel α2δ subunit		
		pregabalin	reduce glutamate		
		Retigabine	Activate potassium channels, induce hyperpolarization	Limited by dizziness, somnolence	[107,108]
		Ketamine	Block NMDA receptors	Refractory pain; limited by hallucinations, hypertension	[110]
Combined Therapy Targeting Microglia-Neuron Crosstalk	Multi-target synergistic analgesia	Minocycline + gabapentin	Inhibit microglia + neuronal excitability	Superior to monotherapy	[106,129]
	Pharmacological + non-pharmacological	Physical therapy	Improve blood circulation, relieve spasm	Adjuvant effect	[114]
		Psychological therapy	Improve mental state, enhance drug efficacy	Improve overall outcome	
		Acupuncture	Modulate neuroimmune network	Regulate microglia and neurons	[116]
Novel Translational Approaches	Gene therapy	IL-10 overexpression; RNAi (IL-1β, TNF-α)	Regulate key gene expression	Preclinical/early clinical	[119,130]
	Cell therapy	Mesenchymal/neural stem cells	Secrete anti-inflammatory/neurotrophic factors	Effective and safe in trials	[121]
	Nanomedicine	Minocycline/gabapentin-loaded nanocarriers	Targeted delivery, improve bioavailability	High targeting, promising translation	[125,127]

## 7. Research Controversies and Challenges

Although significant progress has been made in the research on microglia-neuron crosstalk in chronic pain, there are still many controversies and challenges that need to be addressed. One of the main controversies is the phenotypic classification of activated microglia. The traditional M1/M2 phenotypic classification is too simplistic, and recent studies have shown that activated microglia have significant phenotypic plasticity and heterogeneity, and there are many intermediate phenotypes between M1 and M2 [131]. The specific role of different microglial phenotypes in chronic pain is still unclear, and further research is needed to clarify the phenotypic changes in microglia in different types of chronic pain and their regulatory mechanisms. Another controversy is the specific molecular mechanism of microglia-neuron crosstalk. Although many signaling pathways (such as the pro-inflammatory factor pathway and the purinergic signal pathway) have been identified, the interaction between these pathways and their specific regulatory mechanisms in chronic pain are still not fully clear [48,128]. For example, the crosstalk between BDNF and pro-inflammatory factors in microglia–neuron interaction needs further study [69]. Challenges in translational medicine mainly include the gap between basic research and clinical practice. Many drugs that are effective in animal models of chronic pain have failed in clinical trials [51]. The main reasons include the differences between animal models and human chronic pain, the lack of specific biological markers for patient stratification, and the side effects of drugs [9]. In addition, the long-term efficacy and safety of novel therapeutic approaches (such as gene therapy or cell therapy) need to be further confirmed in clinical trials. The interaction between chronic pain and comorbidities (such as anxiety, depression or cognitive impairment) is also a major challenge. The neuroimmune network imbalance in chronic pain is closely associated with the occurrence of comorbidities, but the specific regulatory mechanism is still unclear [132]. The development of therapeutic strategies that simultaneously relieve chronic pain and its comorbidities is an important direction for future research.

## 8. Conclusions and Future Prospects

In this review systematically, we summarize the research progress on microglia-neuron crosstalk in chronic pain, encompassing the neuroimmune basis, molecular mechanisms, pathological processes, clinical diagnosis, therapeutic strategies, research controversies, and future directions. Chronic pain is a complex pathological state involving intricate interactions between the nervous system and immune system, with the core mechanism centered on the bidirectional regulation of microglial activation and neuronal excitability, as well as neuroimmune network imbalance. The bidirectional crosstalk between microglia and neurons plays a pivotal role in the initiation and maintenance of chronic pain, and targeting this interaction represents a promising direction for the development of novel translational therapeutic strategies.

Notably, chronic pain is characterized by neuroimmune network dysregulation, with microglia–neuron bidirectional communication as its core mechanism—this conclusion identifies the most promising molecular targets for chronic pain intervention supported by evidence in the literature. Microglial P2X4 receptors are a key target: activated by neuronal ATP, they trigger the ERK/p38 MAPK pathway to promote pro-inflammatory factor and BDNF release; blocking ATP release in CCI models reduces P2X4 expression and hyperalgesia, while the P2X4 antagonist TNP-ATP alleviates central sensitization, demonstrating favorable translational potential [132]. The BDNF-TrkB pathway is another vital target: microglia-derived BDNF binds neuronal TrkB receptors, disrupting chloride homeostasis and enhancing neuronal excitability, and microglial BDNF knockout in SNI mouse models alleviates mechanical allodynia [43]; BDNF also promotes microglial activation, forming a pro-nociceptive feedback loop. These targets, closely linked to chronic pain pathogenesis and supported by preclinical evidence, are crucial for developing precise therapies and bridging basic and clinical research in translational medicine. Looking forward, advancing the translational application of basic research findings in clinical practice will focus on several key directions: further clarifying microglial phenotypic heterogeneity and the specific molecular mechanisms of microglia–neuron crosstalk across different chronic pain subtypes using advanced technologies such as single-cell sequencing and spatial transcriptomics; developing specific biological markers for chronic pain diagnosis and patient stratification to facilitate personalized treatment; optimizing existing therapeutic strategies and promoting the clinical translation of novel approaches (e.g., gene therapy, cell therapy, nanomedicine); balancing the inhibition of neuronal excitability and regulation of microglial activation to maximize pain prevention and neuronal function recovery; and strengthening research on the interaction between chronic pain and its comorbidities to develop integrated therapeutic strategies. Despite existing controversies and challenges, with the continuous advancement of basic and translational medicine, more precise and effective therapeutic strategies for chronic pain will be developed, significantly improving patients’ quality of life and reducing the socioeconomic burden.

## Data Availability

No new data were created or analyzed in this study. Data sharing is not applicable to this article.

## Abbreviations

The following abbreviations are used in this manuscript:

Akt	Protein Kinase B
BDNF	Brain-Derived Neurotrophic Factor
CCI	Chronic Constriction Injury
CCL2	C-C Motif Chemokine Ligand 2
CCR2	C-C Motif Chemokine Receptor 2
CSF1	Colony-Stimulating Factor 1
CSF1R	Colony-Stimulating Factor 1 Receptor
CNS	Central Nervous System
CX3CL1	C-X3-C Motif Chemokine Ligand 1
CX3CR1	C-X3-C Motif Chemokine Receptor 1
DAMPs	Damage-Associated Molecular Patterns
ERK	Extracellular Regulated Protein Kinase
EMG	Electromyography
GABA	γ-Aminobutyric Acid
GDNF	Glial Cell Line-Derived Neurotrophic Factor
HPA	Hypothalamic–Pituitary–Adrenal
IL-1β	Interleukin-1β
IL-6	Interleukin-6
IL-10	Interleukin-10
IBA-1	Ionized Calcium-Binding Adapter Molecule 1
JAK/STAT3	Janus Kinase/Signal Transducer and Activator of Transcription 3
KCC2	Potassium-Chloride Cotransporter 2
Kv1.2	Potassium Voltage-Gated Channel Subfamily A Member 2
MAPK	Mitogen-Activated Protein Kinase
MMPs	Matrix Metalloproteinases
MRI	Magnetic Resonance Imaging
NCV	Nerve Conduction Velocity
NGF	Nerve Growth Factor
NF-κB	Nuclear Factor-κB
NLRP3	NOD-Like Receptor Pyrin Domain-Containing 3
NSAIDs	Non-Steroidal Anti-Inflammatory Drugs
NRS	Numerical Rating Scale
NMDA	N-Methyl-D-Aspartate
PET	Positron Emission Tomography
PDI	Pain Disability Index
PI3K	Phosphatidylinositol 3-Kinase
PNN	Perineuronal Net
PAMPs	Pathogen-Associated Molecular Patterns
PRRs	Pattern Recognition Receptors
RNAi	RNA Interference
SF-36	Short Form-36
SNI	Spared Nerve Injury
TLRs	Toll-Like Receptors
TNF-α	Tumor Necrosis Factor-α
TRPV1	Transient Receptor Potential Vanilloid 1
VAS	Visual Analog Scale
VRS	Verbal Rating Scale
Nav1.7	Sodium Voltage-Gated Channel Nav1.7
Nav1.8	Sodium Voltage-Gated Channel Nav1.8
CaV3.2	Calcium Voltage-Gated Channel CaV3.2
P2X3	Purinergic Receptor P2X3
P2X4	Purinergic Receptor P2X4
P2X7	Purinergic Receptor P2X7
P2Y	Purinergic Receptor P2Y
TrkB	Tropomyosin Receptor Kinase B
TrkA	Tropomyosin Receptor Kinase A
TGF-β	Transforming Growth Factor-β
